# Design of an Osteoinductive Extracellular Fibronectin Matrix Protein for Bone Tissue Engineering

**DOI:** 10.3390/ijms16047672

**Published:** 2015-04-07

**Authors:** Sujin Lee, Dong-Sung Lee, Ilsan Choi, Le B. Hang Pham, Jun-Hyeog Jang

**Affiliations:** 1 Department of Biochemistry, College of Medicine, Inha University, Incheon 400-712, Korea; E-Mails: sujin2manse@naver.com (S.L.); ilsan27@hanmail.net (I.C.); plbhang@gmail.com (L.B.H.P.); 2 Department of Biomedical Chemistry, Konkuk University, Chung-Ju 380-701, Korea; E-Mail: dongsunglee@inha.ac.kr

**Keywords:** bone morphogenetic protein, cell adhesion, cell differentiation, fibronectin, MC3T3 cells

## Abstract

Integrin-mediated cell-matrix interactions play an important role in osteogenesis. Here, we constructed a novel osteoinductive fibronectin matrix protein (oFN) for bone tissue engineering, designed to combine the integrin-binding modules from fibronectin (iFN) and a strong osteoinductive growth factor, bone morphogenetic protein-2. Compared with iFN, the purified oFN matrix protein caused a significant increase in cell adhesion and osteogenic differentiation of pre-osteoblast MC3T3-E1 cells (*p* < 0.05).

## 1. Introduction

Fibronectin (FN) is a component of the extracellular matrix and its interaction with integrin is essential in the early stages of osteoblast differentiation [1]. FN binds to the integrin family of cell surface receptors and regulates many biological processes [2]. FN is composed of homologous repeating structural modules consisting of 40–90 amino acids, classified as type I, II, and III repeats (FNI, FNII, and FNIII) [3]. An Arg-Gly-Asp (RGD) sequence located in the tenth type-III domain (FNIII10) and a Pro-His-Ser-Arg-Asn (PHSRN) sequence contained in the ninth type-III domain (FNIII9) of the FN structure represent important motif for cell adhesion through binding with α5β1 integrin receptor, the most common FN receptor [4]. The FNIII9 and FNIII10 modules promote cell adhesion and proliferation, as well as differentiation of osteoblasts [5]. Bone morphogenetic protein-2 (BMP-2), one of the most potent osteoinductive proteins, can accelerate osteoblast differentiation and stimulate the expression of bone matrix proteins such as collagen, osteopontin, and fibronectin [6,7]. BMP-2 stimulates the osteogenic differentiation of pre-osteoblasts into mature osteoblasts by regulating signals that trigger a specific transcriptional program required for bone formation [8,9].

We have previously shown that the integrin-mediated cell adhesion module from FNIII9 and FNIII10 (iFN) synergizes with growth factors to promote cellular responses such as cell adhesion, proliferation, and differentiation [10,11]. The purpose of this study was to construct a novel osteoinductive FN matrix fusion protein (oFN) containing an iFN and an osteoinductive sequence from BMP-2, and evaluate the effects of this new fusion protein on cellular adhesion, proliferation, and differentiation of osteoblastic cells.

Current clinical strategies open involve the combination of proteins with biomaterials such as an absorbable collagen-based carriers, because of the short *in vivo* half-life of proteins. Collagen is the most abundant protein found in the extracellular matrix. Owing to its biocompatibility and biodegradability, collagen is widely used to build scaffold/matrix for biomedical applications [12]. Here, we have further investigated the osteogenic activity of oFN-loaded collagen matrix.

## 2. Results and Discussion

### 2.1. Expression and Purification of oFN Matrix Protein 

**Figure 1:**
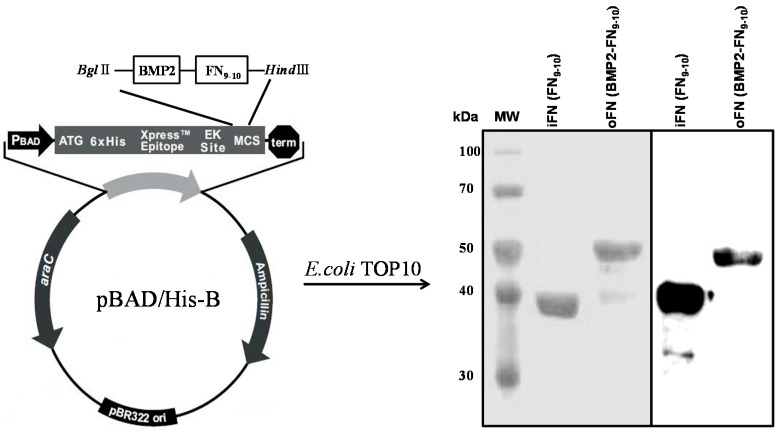
Cloning, expression, and purification of osteoinductive and integrin-binding fibronectin (oFN and iFN, respectively). The cDNA coding for bone morphogenetic protein-2 (BMP-2) and the ninth and tenth type-III fibronectin domains (FNIII_9-10_) was cloned into the pBAD-HisB expression vector. Schematic representations of the fusion proteins and western blotting analysis of iFN (FNIII_9-10_) and oFN (BMP-2-FNIII_9-10_) are shown at 37 and 50 kDa, respectively.

We recently reported that the iFN-osteocalcin fusion proteins are observed in an end-on orientation after absorption from the measurements of dissipation energy (quartz crystal microbalance) and atomic force microscopy (AFM), indicating that the active sites of the iFN-osteocalcin fusion proteins are exposed [13]. Here, we used a similar approach to construct oFN matrix fusion proteins. The oFN was designed to contain the iFN (FNIII9 and FNIII10 modules, the key cell-binding domain of FN) connected to BMP-2. The apparent size of the expressed oFN was in good agreement with the estimated molecular mass, *i.e.*, 50 kDa for a fusion protein consisting of oFN and BMP-2. The hexahistidine-tagged FNs were over 95% pure, as determined by Coomassie Blue staining of 12% (*v*/*v*) SDS-PAGE under reducing conditions. The iFN and oFN bands were also confirmed by western blotting using a peroxidase conjugate of a monoclonal anti-polyhistidine antibody (Figure 1).

### 2.2. Cell Adhesion Activity of oFN Matrix Protein

**Figure 2:**
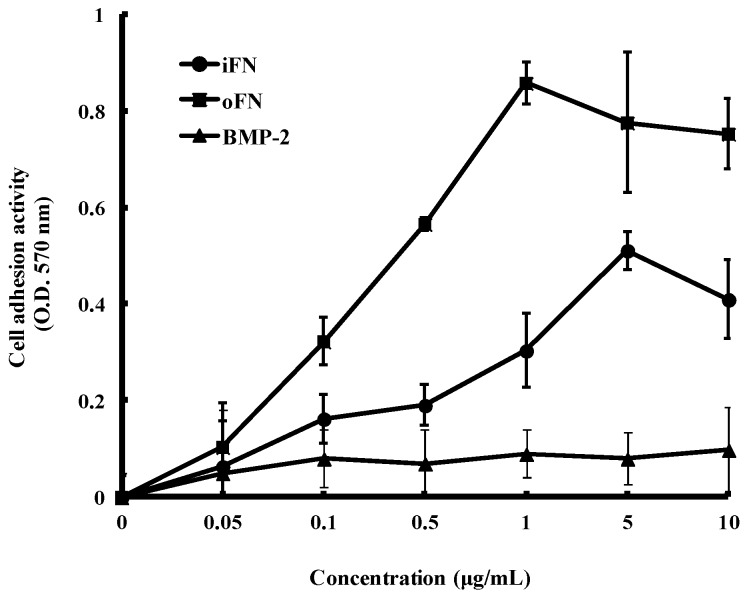
Cell adhesion activity of osteoinductive fibronectin on MC3T3-E1 cells. Cells were seeded at a density of 1 × 10^5^ cells/well on plates coated with iFN, oFN, or BMP-2 and incubated for 30 min at 37 °C. Adhesion of MC3T3-E1 cells was measured as described in Experimental Section. The cell adhesion activity is expressed as absorbance and represented as mean ± standard deviation (*n* = 3).

As observed in our previous study [10], iFN-mediated integrin signaling has been suggested to act with other growth factors signaling pathways to coordinate cellular adhesion. To determine the ability of the oFN matrix protein to enhance cell adhesion compared with iFN, MC3T3-E1 cells were cultured in serum-free medium and then plated on plastic tissue culture dishes coated with iFN or oFN. Figure 2 shows examples of dose-response curves for oFN and iFN. We observed that an increased cellular adhesions of MC3T3-E1 cells plated on oFN-coated substrates, as compared with those on the iFN-coated substrates. However, BMP-2 alone was insufficient to promote significant cellular adhesions of MC3T3-E1 cells. Our data suggest that the oFN matrix protein caused a significant increase in cell adhesion activity compared with the iFN protein. In consistent to our previous studies [10], this synergistic effect of oFN matrix protein on cell adhesion could be due to the coordination between FN-mediated integrin signaling and BMP-2' signaling.

### 2.3. Osteogenic Differentiation Activity of oFN Matrix Protein

**Figure 3:**
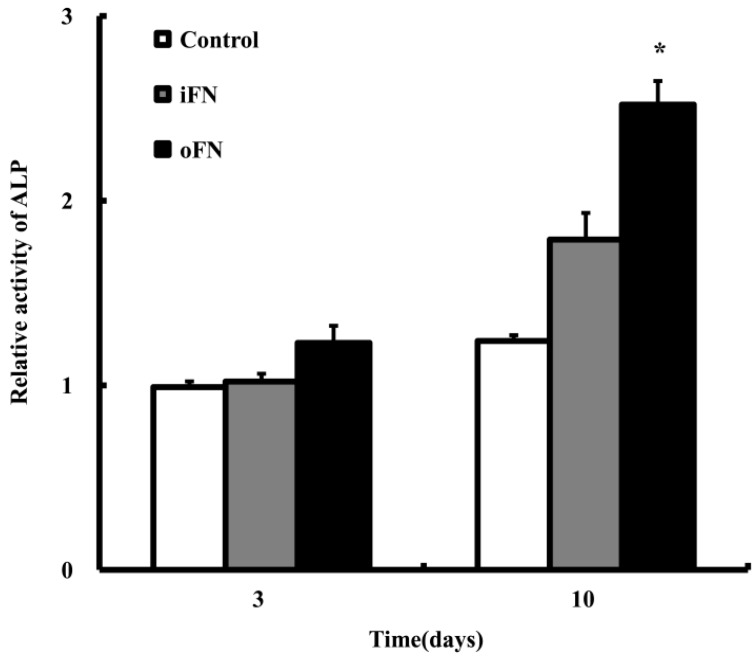
Alkaline phosphatase (ALP) activity of osteoinductive fibronectin (oFN, 5 μg·mL^−1^) on MC3T3-E1 cells, at 3 and 10 days. Cells were seeded at a density of 1 × 10^3^ cells/well on plates coated with integrin-binding fibronectin (iFN) or oFN and incubated. Non-treated cells were used as control. ALP activity was normalized *versus* control and is represented as mean ± standard deviation (*n* = 3). * *p* < 0.05 compared with iFN-loaded groups or control.

The osteogenic differentiation effect of oFN on MC3T3-E1 cells was determined by examining the ALP activity (one of the important early markers for osteogenic differentiation) after culturing the MC3T3-E1 cells on the oFN-coated substrates. The ALP activity in MC3T3-E1 cells coated with oFN was significantly higher compared to that of cells in iFN-coated dishes in culture (Figure 3). These results indicate that the oFN matrix protein caused a 1.5-fold increase at the osteogenic differentiation of MC3T3-E1 cells, compared with that induced by iFN (*p* < 0.001). This increase of ALP activity could be due to BMP-2' signaling from oFN matrix protein.

### 2.4. Effect of oFN-Loaded Collagen Matrix on MC3T3-E1 Cells Differentiation

**Figure 4:**
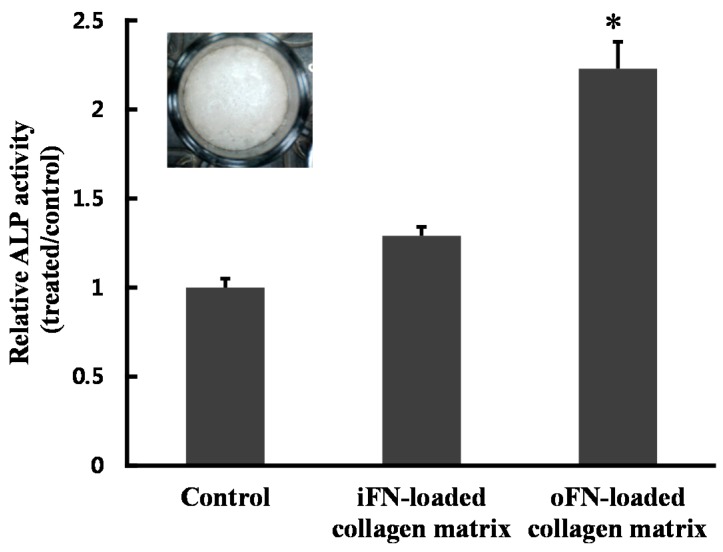
Alkaline phosphatase (ALP) activity in MC3T3-E1 cells seeded on fibronectin-loaded collagen matrix, at 10 days. Collagen matrices were placed in 24-well plates, and MC3T3-E1 cells were cultured on collagen matrices loaded with integrin-binding fibronectin (iFN) or osteoinductive fibronectin (oFN, 5 μg·mL^−1^). Inset: image of a collagen matrix. The results are reported as mean ± standard deviation (*n* = 3). * *p* < 0.05 compared with iFN-loaded groups or control.

Having demonstrated the higher effectiveness of oFN in osteogenic differentiation, the osteogenic ability of MC3T3-E1 cells in direct contact with oFN-loaded collagen scaffolds was then assessed. As shown in Figure 4, the ALP activity of MC3T3-E1 cells was significantly increased on cells grown on the oFN-loaded collagen-based matrices, compared with that on non-treated or iFN-loaded collagen matrices (*p* < 0.05). These results confirm that the oFN-loaded collagen matrices might be significantly more effective in osteogenic differentiation than non-treated or iFN-loaded collagen matrices.

### 2.5. Osteogenic Differentiation Activity of oFN Matrix Protein

**Figure 5:**
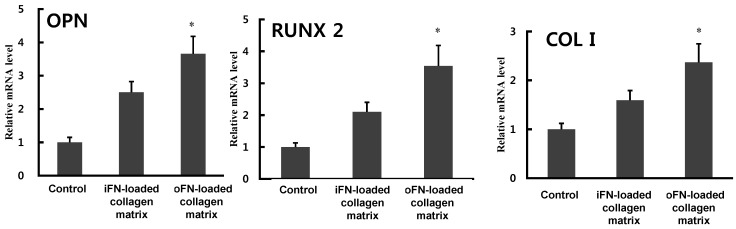
Osteogenic differentiation activity of osteoinductive fibronectin (oFN) matrix protein as determined by real-time PCR. MC3T3-E1 cells were cultured on collagen matrices loaded with integrin-binding fibronectin (iFN) or oFN (5 μg·mL^−1^) in 24-well plates. The mRNA levels of *OPN*, *RUNX2*, and *Col I* were measured, and the relative mRNA level of each gene was normalized to that of GAPDH. The results are reported as mean ± standard deviation (*n* = 3). * *p* < 0.05 compared with iFN-loaded groups or control.

To further confirm the osteogenic differentiation activity of oFN, inductions of osteogenic marker gene expressions, including osteopontin (*OPN*), Runt-related transcription factor 2 (*RUNX2*), and collagen type I (*Col I*), were analyzed by quantitative real-time PCR. Expression levels of all these critical genes in oFN-loaded collagen matrix were significantly upregulated compared to those of control or iFN-loaded collagen matrix (Figure 5), suggesting that oFN could be more effective in osteogenic differentiation. However, the expression of osteocalcin was not significantly induced by oFN. FN-mediated integrin signaling synergizes with signals from growth factors to promote cellular adhesion and differentiation in osteoblast cells [10,14]. In the present study, we described the construction of a novel osteoinductive fibronectin matrix fusion protein, which combines integrin-binding modules from fibronectin and BMP-2. Cell adhesion to FN triggers a number of intracellular signalings. This FN-mediated integrin signaling has been suggested to act with other growth factors signaling pathways to coordinate cellular adhesion, proliferation, and differentiation [15]. Consistently with the previous studies, the engineered oFN induced significantly better cellular adhesion and osteogenic differentiation compared to that observed with iFN only.

## 3. Experimental Section 

### 3.1. Construction of the Osteoinductive FN Matrix Protein

In order to construct the osteoinductive FN, the osteoinductive domain of the BMP-2 sequence (Gln283-Arg396) was generated by polymerase chain reaction (PCR) amplification, using the primers 5'-CCAGATCTCAGGCGAAACATAAACAG-3' (which introduces a *Bgl*II restriction site (indicated by underline) before the NH_2_-terminus of BMP-2) and 5'-AAGGTACCACGGCAGCCGCAGCCTTC-3' (which incorporates a *Kpn*I restriction site (indicated by underline) at the COOH-terminus of BMP-2). PCR was performed in 30-µL reaction mixes containing 50 mM KCl, 10 mM Tris-HCl (pH 8.3), 1.5 mM MgCl_2_, 100 µg·mL^−1^ gelatin, 0.2 mM deoxynucleotide triphosphates (dNTPs), 1.25 U *Taq* polymerase (iNtRON, Seoul, Korea), and 50 pmol each of the forward and reverse primers. PCR involved 30 cycles of 1 min at 55 °C (annealing), 1 min at 72 °C (extension), and 1 min at 94 °C (denaturation). The amplified products were digested with *Bgl*II and *Kpn*I. After digestion, the PCR products were in-frame ligated into the *Bgl*II *and Kpn*I sites of pBAD/HisB/iFN [16].

### 3.2. Protein Expression and Purification

To express iFN and oFN, *E. coli* TOP10 cells were grown overnight in Luria-Bertani medium with ampicillin (LB-Amp) at 37 °C. When the absorbance at 600 nm (A_600_) of the cultures reached 0.6, induction was initiated with 0.25% (*w*/*v*) l-arabinose. Three hours later, bacteria were pelleted by centrifugation at 6000× *g* for 10 min, lysed, and sonicated. A soluble extract was prepared by centrifugation at 14,000× *g* for 30 min in a refrigerated centrifuge and the resulting supernatant was transferred to a fresh tube. The crude protein obtained from the sonicated bacterial supernatant was purified via binding of a hexahistidine tag (located at the amino-terminal end of iFN) to a nickel-nitrilotriacetic acid resin column, according to the manufacturer’s instructions (Invitrogen, Carlsbad, CA, USA). The degree of purification of the recombinant protein was determined under denaturing conditions by Coomassie blue staining of 12% (*v*/*v*) SDS-PAGE gel. Western blots were performed using a peroxidase conjugate of a monoclonal anti-polyhistidine antibody (sc-8036, Santa Cruz Biotechnology, Santa Cruz, CA, USA) to confirm the expression of the recombinant fusion protein. The molecular size of the immunodetected protein was identified by comparing its mobility to that of pre-stained protein markers (Elpis Biotech, Daejeon, Korea) electrophoresed in parallel lanes.

### 3.3. Cell Culture

The MC3T3-E1 cell is a pre-osteoblastic cell line established from newborn mouse calvaria. MC3T3-E1 cells were cultured in α-minimum essential medium (α-MEM, Invitrogen, Carlsbad, CA, USA) containing 10% heat-inactivated fetal bovine serum (Invitrogen, Carlsbad, CA, USA), 100 U·mL^−1^ penicillin G sodium, 100 μg·mL^−1^ streptomycin sulfate, and 0.25 μg·mL^−1^ amphotericin B (Invitrogen, Carlsbad, CA, USA) in a 5% CO_2_ atmosphere at 37 °C. Confluent cells were detached with 0.25% trypsin-EDTA for 5 min, and aliquots were subcultured. MC3T3-E1 cells maintained for three passages were used for further cell proliferation and differentiation studies.

### 3.4. Cell Adhesion Assay

The cell adhesion activity was measured using the crystal violet assay. 24-well plates were coated with either iFN or oFN by adsorption overnight at 4 °C. The iFN and oFN concentrations for the coating were 0.05, 0.1, 0.5, 1, 5, and 10 μg·mL^−1^. Each sample was assayed in triplicate wells. After incubation, each well was washed with Dulbecco’s phosphate buffered saline (DPBS), and the wells were then blocked with 1% (*w*/*v*) BSA solution for 30 min. MC3T3-E1 cells, prepared in α-MEM serum-free medium at 5 × 10^3^ cells/well, were seeded in each plate. To calculate the adsorbed amount of protein, the amount of proteins in the supernatant was measured by BCA protein assay (Pierce). The 99% of loaded iFN and oFN was adsorbed after overnight at 4 °C. After incubation for 30 min at 37 °C, adherent cells were washed twice with DPBS, and fixed with 3.7% (*w*/*v*) formalin solution for 15 min at room temperature. The cells were stained with 0.25% (*w*/*v*) crystal violet (Sigma, St. Louis, MO, USA) in 2% (*v*/*v*) ethanol/water for 1 h at 37 °C and then gently washed three times with DPBS. For cell lysis, 2% sodium dodecyl sulfate (SDS) solution was added and transferred into 96-well plates. The absorbance at 570 nm was measured using a microplate reader (Molecular Devices, Menlo Park, CA, USA).

### 3.5. Cell Differentiation Assay

To assess alkaline phosphatase (ALP) activities, MC3T3-E1 cells were plated at 1 × 10^3^ cells/well for 3 and 10 days with 5 μg·mL^−1^ iFN or oFN, and rinsed with DPBS and lysed with 200 μL 1% Triton X-100. Each cell suspension was mixed with 100 μL *p*-nitrophenyl phosphate (*p*-NPP). *p*-NPP, which is yellow in color, was produced in the presence of alkaline phosphatase, and its absorbance at 405 nm was determined spectrophotometrically using a microplate reader (BioRad Laboratories, Hercules, CA, USA). The ALP activity was normalized to the control. For ALP assay of iFN or oFN-loaded collagen matrix, collagen matrices were placed in 24-well plates. Collagen matrix was prepared as described previously [12]. Briefly, Collagen was ground in a mortar, dissolved in distilled water, and cross-linked using 1-Ethyl-3-(3-dimethylaminopropyl)carbodiimide hydrochloride (EDC). Then, 500 μL of collagen sol was distributed to each well of 24-well plates and freeze-dried. The freeze-dried collagen matrices were washed with phosphate-buffered saline (PBS) solution several times before use. The 99% of loaded iFN and oFN was adsorbed after overnight at 4 °C.

### 3.6. RNA Extraction and cDNA Synthesis

Total RNA was extracted using an Easy-spin RNA extraction kit (iNtRON, Seoul, Korea). The RNA purity was assessed by the absorbance at 260 and 280 nm (A_260_/A_280_ ratio values of 1.9–2.1 were considered acceptable) and by ethidium bromide staining of 18S and 28S RNA on gel electrophoresis. RNA concentrations were determined from A_260_ values. Two µg of total RNA was reverse-transcribed in a 20 μL reaction mixture containing 50 U of SuperScript II reverse transcriptase (Invitrogen, Carlsbad, CA, USA), 5 μm DTT, 40 U of RNaseOUT recombinant ribonuclease inhibitor, 0.5 μm random hexanucleotide primers, and 500 μm dNTP mixture. Reverse transcription was carried out at 50 °C for 60 min. The reaction mixture was subsequently heated at 70 °C for 15 min to terminate the reaction. The cDNA product was stored at −20 °C.

### 3.7. Quantitative Real-Time PCR Analysis

The over-expressions of the obtained genes were confirmed by quantitative real-time PCR. All real-time PCR analyses were performed on an ABI Step One real-time PCR system. Each reaction was performed in a 20-μL reaction mixture containing 0.1 μm of each primer, 10 μL of 2× SYBR Green PCR master mix (Applied Biosystems, including AmpliTaq Gold DNA polymerase in buffer, a dNTP mix, SYBR Green I dye, ROX dye, and 10 mM MgCl_2_), and 1 μL of template cDNA. PCR was conducted by activation of the enzyme at 94 °C for 10 min followed by 40 cycles of denaturation at 94 °C for 15 s, annealing at 60 °C for 1 min, and extension at 60 °C for 1 min. The *C*_t_ (cycle threshold) value for each gene, determined using the automated threshold analysis in the ABI instrument, was normalized with respect to *C*_t(GAPDH)_ to obtain d*C*_t_ = *C*_t(GAPDH)_ − *C*_t(specific gene)_. A difference *n* between two *C*_t_ or d*C*_t_ values denoted a 2^n^-fold difference in the target sequence between the two cDNA samples being compared. The primers used for quantitative PCR are reported in Table 1.

**Table 1:** Sequences of the primers used in the real-time polymerase chain reaction (PCR).

Genes	Forward Primer	Reverse Primer
*Runx2*	GGCCGGGAATGATGAGAACTA	GGCCCACAAATCTCAGATCGT
*OPN*	CCAATGAAAGCCATGACCAC	CGACTGTAGGGACGATTGGA
*Col І*	GAAAGGATCTCCTGGTGCTG	ACCTTGTTTGCCAGGTTCAC
*GAPDH*	TCCACTCACGGCAAATTCAAC	AGCCCAAGATGCCCTTCAGT

### 3.8. Statistical Analysis

Experimental results were expressed as the mean ± standard deviation (SD). The statistical analyses were performed using one-way ANOVA (* *p* < 0.05). In all statistical analyses, a *p* value below 0.05 was considered as significant.

## 4. Conclusions

In conclusion, we developed a novel osteoinductive FN matrix protein that was able to enhance both cell-adhesion activity and osteoinduction. The engineered oFN matrix fusion protein resulted in more effective bone regeneration by promoting cellular adhesion and differentiation. Thus, the present work shows that design of fusion proteins could represent a highly relevant approach for bone tissue engineering.
